# Three-dimensional muon imaging of cavities inside the Temperino mine (Italy)

**DOI:** 10.1038/s41598-022-26393-7

**Published:** 2022-12-25

**Authors:** Diletta Borselli, Tommaso Beni, Lorenzo Bonechi, Massimo Bongi, Debora Brocchini, Nicola Casagli, Roberto Ciaranfi, Luigi Cimmino, Vitaliano Ciulli, Raffaello D’Alessandro, Andrea Dini, Catalin Frosin, Giovanni Gigli, Sandro Gonzi, Silvia Guideri, Luca Lombardi, Massimiliano Nocentini, Giulio Saracino

**Affiliations:** 1 Department of Physics and Astronomy, University of Florence, 50019 Florence, Italy; 2 Department of Physics and Geology, University of Perugia, 06123 Perugia, Italy; 3 INFN Florence Division, 50019 Florence, Italy; 4 Parchi Val di Cornia S.p.A., 57025 Piombino, Italy; 5 Department of Earth Sciences, University of Florence, 50121 Florence, Italy; 6 Department of Physics Ettore Pancini, University of Naples Federico II, 80126 Naples, Italy; 7 INFN Naples Division, 80126 Naples, Italy; 8 CNR, Institute of Geosciences and Georesources, 56127 Pisa, Italy

**Keywords:** Applied physics, Experimental particle physics, Imaging techniques, Geophysics, Natural hazards

## Abstract

Muon radiography (muography) is an imaging technique based on atmospheric muon absorption in matter that allows to obtain two and three-dimensional images of internal details of hidden objects or structures. The technique relies on atmospheric muon flux measurements performed around and underneath the object under examination. It is a non-invasive and passive technique and thus can be thought of as a valid alternative to common prospecting techniques used in archaeological, geological and civil security fields. This paper describes muon radiography measurements, in the context of archaeological and geological studies carried out at the Temperino mine (LI, Tuscany, Italy), for the search and three-dimensional visualisation of cavities. This mine has been exploited since Etruscan times until recently (1973), and is now an active tourist attraction with public access to the tunnels. Apart from the archaeological interest, the importance of mapping the cavities within this mine lies in identifying the areas where the extraction ores were found and also in the safety issues arising from the tourist presence inside the mine. The three-dimensional imaging is achieved with two different algorithms: one involving a triangulation of two or more measurements at different locations; the other, an innovative technique used here for the first time, is based on the back-projections of reconstructed muon tracks. The latter requires only a single muographic data tacking and is to be preferred in applications where more than one site location can be difficult to access. Finally the quality of the three-dimensional muographic imaging was evaluated by comparing the results with the laser scan profiles obtained for some known cavities within the Temperino mine.

## Introduction

### Muography as an imaging technique

Muography^1^ is a non-invasive imaging technique based on atmospheric muon absorption in matter and can be used to obtain two and three-dimensional images of the inner parts of a *target* structure. The principle of operation is the same as X-ray radiography, but, thanks to the great penetrating power of cosmic muons^2,3^, it can be applied to large structures such as pyramids^4^, but also to very large ones, of the size of a few kilometres, such as volcanoes^5,6^.

Muons are unstable particles, with a mean life of 2.2$\,\mathrm {\mu s}$ and mass about 200 times greater than that of the electron and are the most common charged cosmic particles at sea level. Atmospheric muons are produced in copious amounts from the interaction of cosmic rays with the atmosphere (vertical flux $\sim 70\,\, \mathrm {s^{-1}m^{-2}sr^{-1}}$ for energies greater than 1 GeV). The muon flux at the Earth’s surface depends on the altazimuth angles $(\varphi ,\theta )$, with the flux decreasing with a dependence on the zenith angle $\propto \mathrm {cos^{n}\theta }$ (*n* depending on the energy, i.e. $n=2$ for $E\sim 3\,\textrm{GeV}$)^2^, and only the weak dependence on the azimuth angle $\varphi$ caused by the East-West effect due to the Earth’s magnetic field.

The images obtained with muon radiography, depict the muon flux change, due to muons absorption, through different sections of the target. These images, represent the muon transmisssion, can than be converted into density maps through the comparison with simulations, thus obtaining the angular distribution of the average density along the line of sight of the materials that compose the target. The technique can be applies to many fields: geophysics, archaeology, civil engineering and nuclear safety, with the characteristic of being non-invasive which often makes it a valid alternative to common geophysical monitoring techniques. Muography requires the measurement of the number of muons arriving from a certain angular direction ($\varphi ,\theta$) downstream of the target structure, to which purpose charged particle trackers are employed sometimes in combination with very simple absorber layers to reject potential low-energy backgrounds particles.

In more detail, the methodology used to generate a two-dimensional density image of the inner structure of the target consists of *three steps*. The *first* is the *target configuration* in which the measurement is performed with the detector oriented towards the target structure. From this measurement, the number of muon counts $N_{meas}(\varphi ,\theta )$ for each direction that falls within the detector’s acceptance is obtained. This quantity depends on the target structure, on the cosmic ray flux (which in turn depends mainly on the azimuth angle), and on the efficiency of the detector. These last dependencies can be in most part eliminated by performing a *second step* using a *free-sky configuration* (where there are no structures in front of the detector), with the detector in the same orientation as in the target configuration. Comparing these two measurements, the muon transmission, defined as the fraction of muons that reach the detector in the target configuration with respect to the case of the free-sky configuration, is obtained as^7^:

1 $$\begin{aligned} t_{meas}(\varphi ,\theta )=\dfrac{\Phi _{meas-target}(\varphi ,\theta )}{\Phi _{meas-freesky}(\varphi ,\theta )}\quad \textrm{with}\quad \Phi _{meas}(\varphi ,\theta )=\dfrac{N_{meas}(\varphi ,\theta )}{t_{acq}\,S_{eff}(\varphi ,\theta )} \end{aligned}$$

where $\Phi (\varphi ,\theta )$ is the muon flux measured in the direction identified by the angles $(\varphi ,\theta )$. The flux depends on the number of muons detected in the direction $(\varphi ,\theta )$, on the acquisition time ($t_{acq}$) and on the effective surface of the detector ($S_{eff}(\varphi ,\theta )$) in the direction $(\varphi ,\theta )$. The effective surface is defined as: $S_{eff}(\varphi ,\theta , \nu )=A(\varphi ,\theta )\cdot \varepsilon (\varphi ,\theta , \nu )\cdot S$ where *A* is the detector’s acceptance, *S* is the detector’s active surface and $\varepsilon$ is the efficiency which takes into account the trigger efficiency and the reconstruction efficiency.

To derive the average density of the materials crossed by the muons, the measured transmission must be compared with the simulated ones (*third step*). The simulation must be based an accurate model reproducing the differential flux of cosmic ray at the Earth’s surface, and the known geometry of the target. By varying the target density $\rho$ in the simulation, a number of simulated transmission $t_{simu}(\varphi ,\theta ,\rho )$ are obtained. Comparing this simulated transmission files to the measurement one in the direction ($\varphi ,\theta$), a density ${\overline{\rho }}(\varphi ,\theta )$ can be associated to each measured transmission for each angular direction obtaining a two-dimensional map of the average density along the line of sight of the observed target.

## Methods: three-dimensional reconstruction algorithms

The angular density map gives only two-dimensional information about the target density distribution. From this it is possible to observe, for example, compact areas named *anomalies* with densities significantly different from those of adjacent materials but with no indication of the actual distance of these anomalies from the detector. This additional information can be acquired either through triangulation (involving more than one muographic measurement) or using a different algorithm called *back-projection*^8–11^.

### Triangulation technique

**Figure 1:**
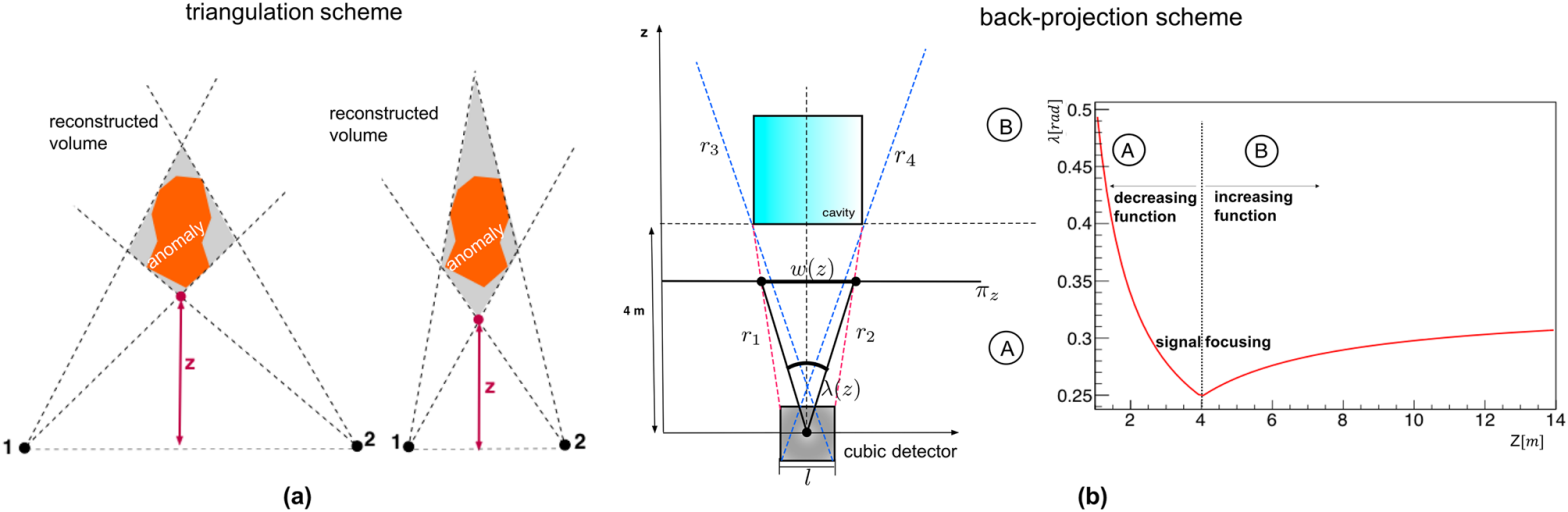
Schematic representation of the three-dimensional reconstruction methods: triangulation (**a**) and back-projection (**b**). In (**a**) the two sketches show the triangulation results of the same object assuming different measurement configurations; the resulting different elongated shapes reconstructed with this technique are shaded in gray. In (**b**) the operating principle of the back-projection technique is schematically represented. The angular size $\lambda (z)$ of cavity seen from the center of the detector reaches its minimum on the plane of back-projection $\pi _{z}$ at the distance at which the base of the cavity is located. At this distance, the transverse linear dimension *w*(*z*) of the cavity on $\pi _{z}$ plane represents the true size. In the figure on the right the analitycal trend of the $\lambda (z)$ function is shown.

The triangulation technique requires at least two measurements of the same target with the detector placed in different positions. Once the density maps have been created from each measurement position and after having identified an anomaly in these maps, it is possible to determine the distance of the anomaly from the detector. As shown in Fig. 1a, from each position the anomaly is seen within a certain cone. The three-dimensional development of the anomaly is obtained from the intersection of the cones into which the anomaly falls, seen from each measurement position. A better vertical resolution can be obtain, by having the measurement positions distant enough from each other. However, it is not always possible to carry out multiple measurements of the same target from different positions, for example the site could be difficult to access or too narrow for the installation of the apparatus. In addition, multiple measurements require either more detectors, longer acquisition time or both. In these situations, for relative near targets (see below), information on the three-dimensional localization of the anomaly can be obtained with a single measurement using the back-projection technique.

### Back-projection technique

The back-projection technique is based on a stereoscopic effect of image generation: two different points on the surface of the same detector see the anomaly from two different angulations, so it is possible to create an internal triangulation in order to locate the anomaly’s structure within the target. The application of the back-projection method requires that some general geometric conditions be satisfied:

- the entire anomaly must be inside the detector’s acceptance;
- the distance between detector and the anomaly must be less than $l/\sigma _{\theta }$, where *l* is related to the detector’s transverse dimensions and $\sigma _{\theta }$ is the detector’s angular resolution (in radians).

The principle of operation of this technique is shown in Fig. 1b in which a cubic cavity is located at a distance of 4 m from the detector. The excess of tracks due to the presence of the cavity compared to the case in which the material is homogeneous falls inside the region between the colored dotted lines ($r_{1}$, $r_{2}$, $r_{3}$, $r_{4}$). The plane $\pi _{z}$ is orthogonal to the pointing direction of the detector and its positions along the *z* axis can be varied over all $z>0$ values. This plane is called the *back-projection plane* and the transverse linear size of the signal region projected to that plane is indicated with *w*(*z*). The corresponding function $\lambda (z)$ represents the angular size of the cavity observed from the centre of the detector and can be expressed as:

2 $$\begin{aligned} \lambda (z)=2\,\textrm{tan}^{-1}\Big (\dfrac{w(z)}{2\,z}\Big ) \end{aligned}$$

In Fig. 1b (on the left) we can distinguish two signal regions: region A, for *z* between detector and the target, defined by the lines $r_{1}-r_{2}$ and region B, for *z* above the target, defined by the lines $r_{3}-r_{4}$. It is demonstrated^8–11^ that the $\lambda (z)$ function has a different behavior in the two signal zones: in A it is a decreasing function and in B it is an increasing function. The change of slope corresponds to the configuration in which the back-projection plane lies on the face of the cavity that looks at the detector. Figure 1b (on the right) shows the analitycal trend of equation (2) obtained from algebraic calculations^11^. A focalization of the signal is observed when the function $\lambda (z)$ reaches a minimum value for $z=4$ m which corresponds to the cavity position. At this distance, the transverse linear dimension *w*(*z*) of the cavity on $\pi _{z}$ plane represents the true size.

In the next chapters the application of these three-dimensional images techniques to the localization of cavities in the Temperino mine are described.

## The MIMA detector and the Temperino mine complex

### The MIMA muon tracker

The muon hodoscope used for the measurements described in this paper is the MIMA^12,13^ detector (***M***uon ***I***maging for ***M***ining and ***A***rchaeology). MIMA was designed by the muon radiography group of INFN and University of Florence and is an evolution and improvement of the MURAY^5^ detector. In particular it is equipped with the front-end electronics and DAQ developed for the MURAVES^14,15^ (***MU***on ***RA***diography of ***VES***uvio) detector. The MIMA detector was assembled and calibrated between the end of 2016 and the first half of 2017 and has performed muography measurements in various contexts, such as geology^7^, archaeology^16^ and civil engineering^17^.

**Figure 2:**
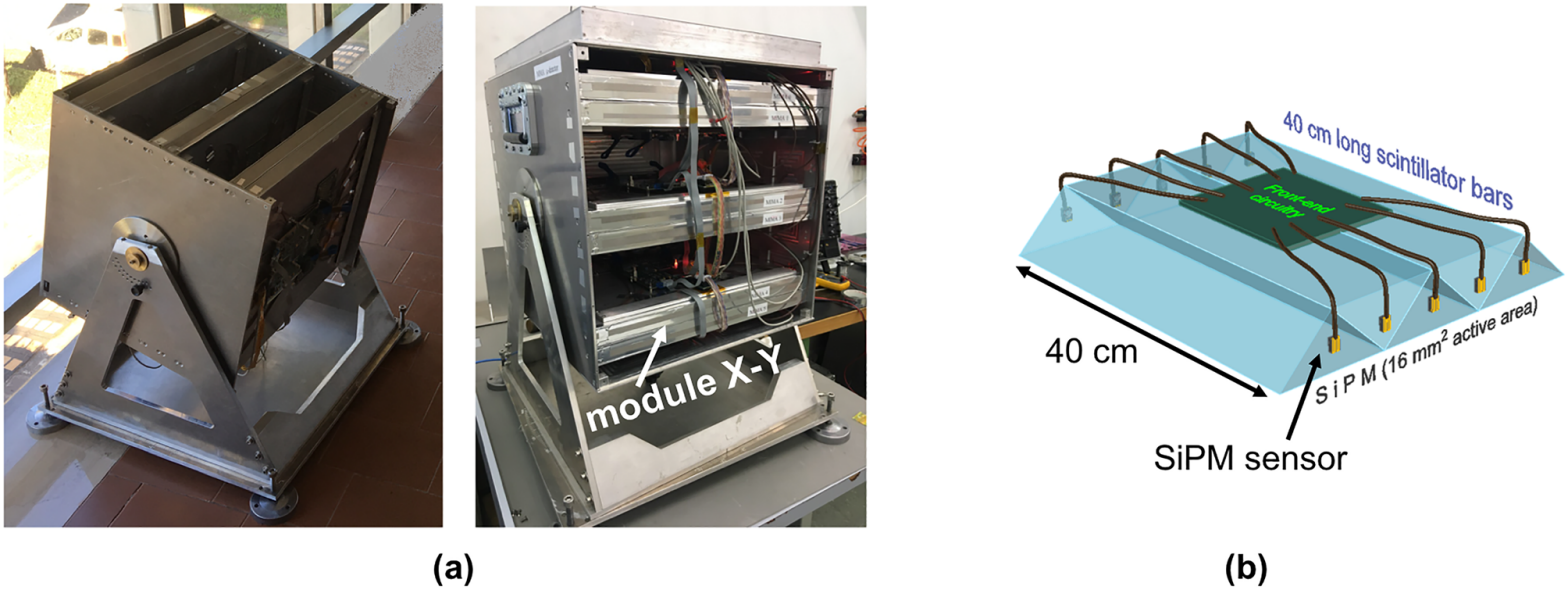
The MIMA muon tracker employed for the muon radiography measurements in the Temperino mine. In (**a**) the detector with the structure of 3 $X-Y$ module of tracking planes. In (**b**) the structure of a single plane consisting of triangular scintillator bars (for simplicity only five bars) is shown^1^.

The MIMA detector (Fig. 2), enclosed in an aluminium protective cover, appears like a cube with an approximate size of $(50 \times 50 \times 50) \, \mathrm {cm ^ {3}}$ and is mounted on a rotating support which allows altazimuth orientation. The detector itself consists of a total of 6 tracking planes arranged in orthogonal pairs, to form 3 compact $X-Y$ modules. Each tracking plane has an active surface of $(40 \times 40)\,\mathrm {cm ^ {2}}$ and consists of 21 plastic scintillators, $40\,\textrm{cm}$ long and with a triangular section of $4 \, \textrm{cm}$ at the base and $2\,\textrm{cm}$ in height. The light signal from each scintillator bar is collected by two SiPM (*Silicon PhotoMultiplier*) which are optically coupled to the scintillator on the two triangular end faces. Details of the SiPMs used, the procedure for digitizing the analogue signals coming out of the SiPM and the data acquisition are found in previous works^12,13^. Assuming that the signal in each bar is proportional to the path travelled by the particle crossing the bar, the triangular shape of the bars allows to use a centre of gravity algorithm to improve the resolution of the reconstructed impact point. The achieved $1.6 \, \textrm{mm}$^18^ spatial resolution, corresponds to an angular resolution of about 7 mrad for the track reconstructed with the whole hodoscope. The features of the MIMA detector make it an apparatus that can be easily transported and installed in small and narrow places such as tunnels or cavities. Thanks to its low power consumption, it does not require special powering systems and can be powered, if required, even by means of a small photovoltaic system.

### The Temperino mine and the MIMA measurement positions

**Figure 3:**
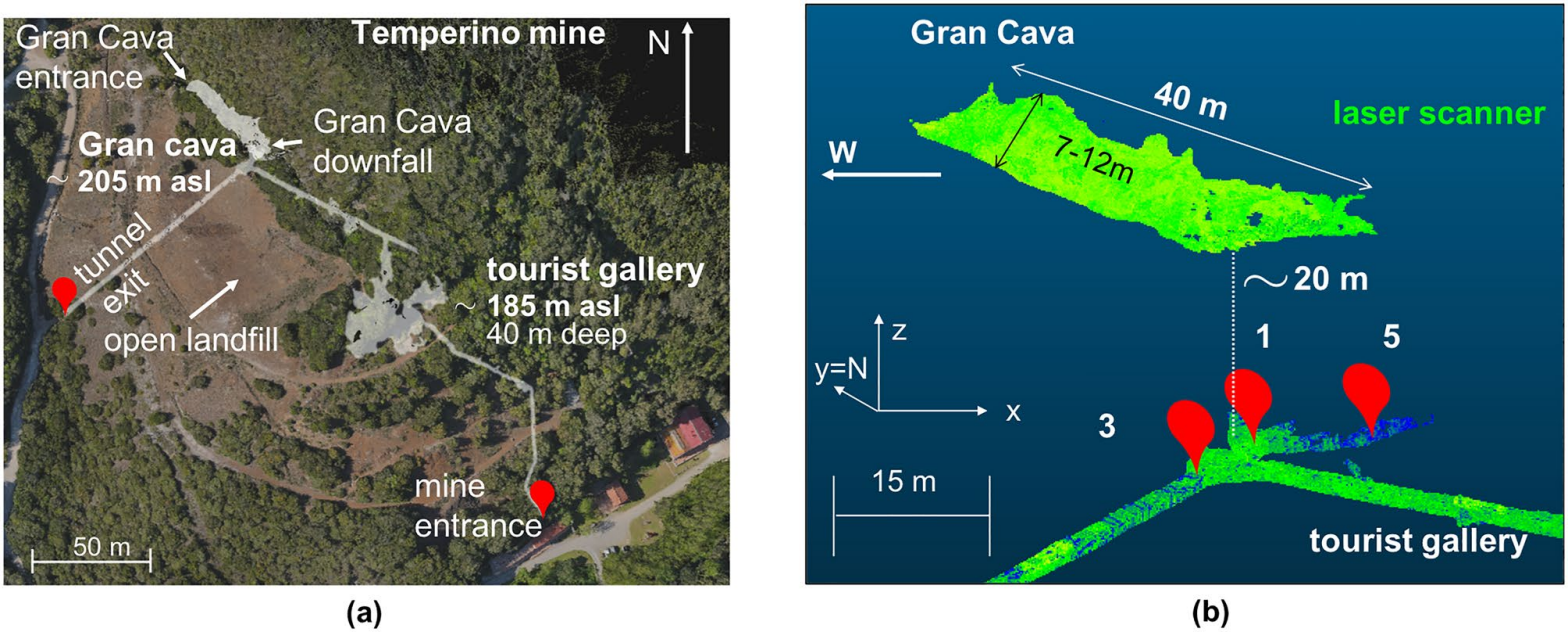
(**a**) the top view of the area of the Temperino mine (LI) in Tuscany obtained from a drone point cloud acquired for this study. The point cloud is displayed in CloudCompare^21^ software. In transparency the gallery opened to the public (located about 40 m deep) and the Gran Cava, a known cavity, 20 m higher than the tunnel are shown. (**b**) localization of the measurement positions inside the tourist gallery. The figure shows the adopted three-dimensional Cartesian reference system with the positive direction of the y axis coinciding with the North direction.

The Temperino mine is located within the Archaeological Mines Park of San Silvestro in the countryside of Campiglia Marittima (LI) in Tuscany. The first mining activities date back to the Etruscan era and manifest themselves with small and tortuous tunnels and vertical wells. There is evidence of activities also from the medieval and post-medieval period up to the most recent that date to the late nineteenth century, when the mine activity ceased completely. The mining activity has always focused on the search for *skarn*, a high density silicate rock containing Cu-Fe-Zn-Pb-Ag sulfides. Skarn in the area occurs as sub-vertical lenses, trending NW-SE, hosted by marbles deposits^19,20^. Skarn bodies are several hundred meters long and few tens of meters thick. The tunnels in the mine have been dug to intercept the clusters of skarn. The mine therefore looks like a myriad of tunnels that are arranged in 6 levels down to about 200 m. Figure 3a shows the drone point cloud, acquired for this study, of the area where the Temperino mine is located and, in transparency, a reconstruction by a 3D laser scanner of the tourist access tunnel, about 360 m long and located at a depth of 40 m from the surface. A large cavity called “Gran Cava”, located 20 m above the tunnel is also shown.

Since 2018 many data collection campaigns have been carried out inside the Temperino mine. This work reports on the results from three measurement positions indicated in Fig. 3b with the numbers 1, 3 and 5. The numbers indicate the chronological order of the measurements. These muographic measurements have a twofold aim: the validation of muography applied to geology through the localization and imaging of known cavities and the investigation of unmapped cavities and mineral deposits. The detector has been installed inside the tourist gallery pointing in a vertical direction, in order to observe the Gran Cava quarry, which represents the known test cavity, with a length about 40 m, width 6-7 m, height 6-7 m. The images shown in Fig. 3b are obtained through point clouds acquired with a laser scanner and exported to the *CloudCompare*^21^ software. The Gran Cava has two external entrances: the main one is in the NW direction and is accessible via a beaten road, the other one called “downfall zone” is in the SE direction and is of uncertain origin (probably due to a collapse). In addition to these external accesses, the Gran Cava is connected via other internal tunnels, some of which are no longer accessible, to the upper and lower floors of the mine.

## Results: imaging and three-dimensional reconstruction of cavities at the Temperino mine

### Muon imaging at the Temperino mine

**Figure 4:**
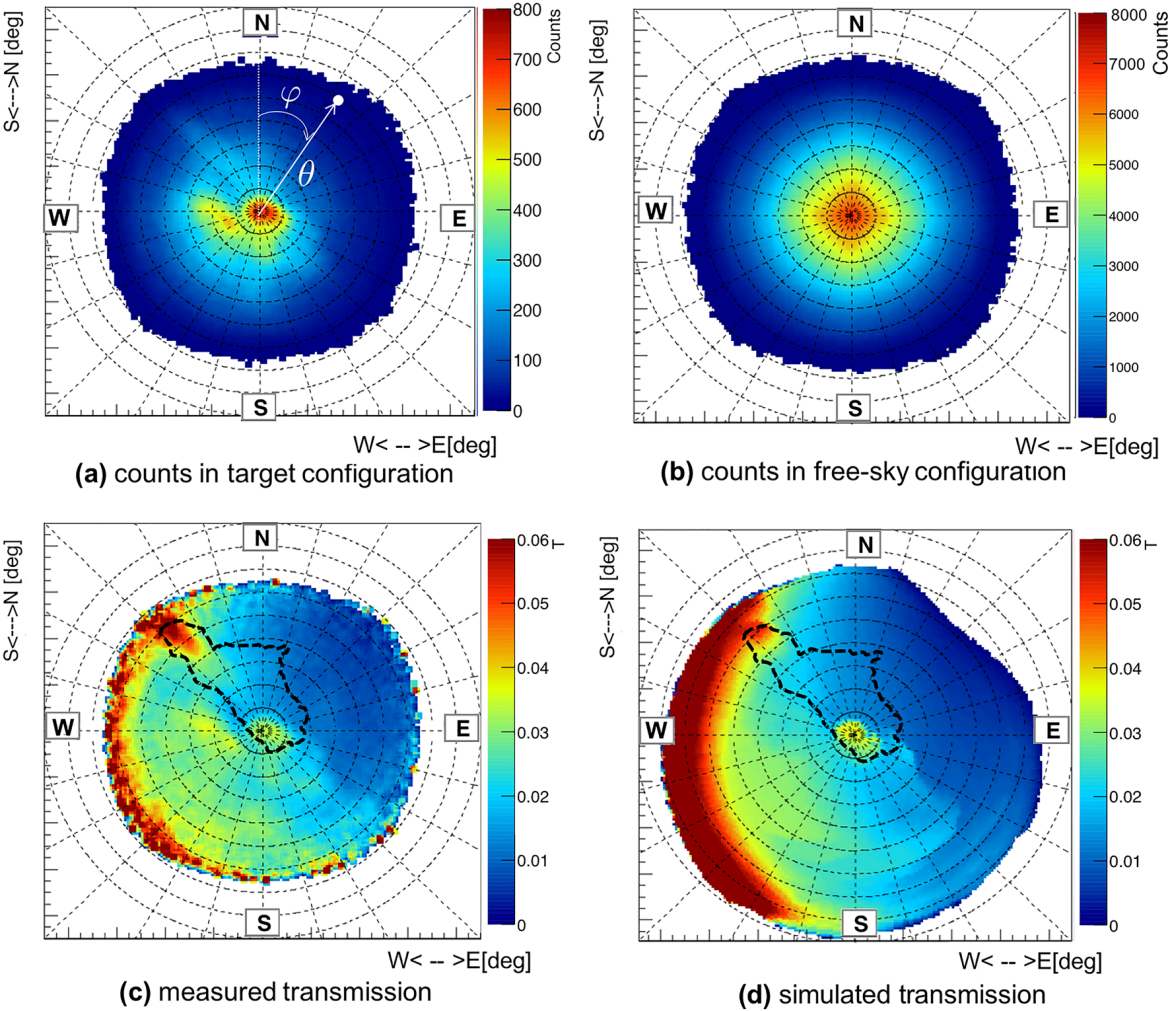
(**a**) the angular distribution of muon counts obtained from installation 1 in the mine (*target configuration*); (**b**) the distribution of the counts obtained in the *free-sky configuration*. (**c**) the measured muon transmission and (**d**) simulated transmission for a homogeneous *target* density of $2.65 \, \mathrm {g / cm ^ {3}}$. In (**c**) and (**d**) the profile of the Gran Cava is shown as a dashed line. ($\varphi$). The acceptance in the simulation (**d**) is larger than that of the detector shown in the other subfigures. The figures show a grid: the circles every 10 degrees represent the zenith angles ($\theta$), the lines every 15 degrees represent the azimuth angles. The images are obtained with the ROOT^22^ framework.

In order to obtain the measured transmission $t_ {meas} (\varphi , \theta )$ of muons at the measurement positions it is necessary to compare the muon counts obtained in the target configuration with those measured in a free-sky one. The two-dimensional angular maps of the counts obtained in both configurations are shown in Fig. 4a which refers to measurement position 1 (similar maps are obtained for measurement position 3 and 5) and in Fig. 4b. As indicated in Fig. 4a, each bin represents a particular pseudo-polar direction $(\varphi , \theta )$, the value of the zenith angle $\theta$ is the distance from the centre and the value of the azimuth angle $\varphi$ is evaluated clockwise from North. The center of the map represents the vertical direction of the detector (corresponding to $\theta =0^{\circ }$). These maps are geo-referenced and must be interpreted as seen from above. The measurements in the mine lasted about 60 days, for a total of about 2 million reconstructed events. The detector was oriented towards the zenith, under an overburden of roughly fifty metres of rock, with a resulting trigger rate of about 0.5 Hz and with each $X-Y$ module achieving more than 99% efficiency. Figure 4b refers to a free-sky measurement made previously at the INFN building in Florence with the detector again pointing to the zenith. In this case, about 37 million reconstructed events were acquired in 53 days with a trigger rate of about 22 Hz.

Using equation (1) we obtain the plot in Fig. 4c, which represents the muon measured transmission $t_ {meas}(\varphi ,\theta )$ in the mine from measurement position 1, with the contour of the Gran Cava’s laser scan shown. In correspondence of the two access points of the quarry there is a noticeable increase in the muon transmission. Furthermore, coherently with the morphological trend of the hill, the muon transmission decreases in the SW-NE direction, due to the increase in the rock layer’s thickness. The simulation program used in this work^7,23^ includes a realistic ground muon flux model and implements the geometry of the target structure (i.e. the Gran Cava). Concerning the geometry of the mine and the thickness of the rock crossed, we used a LiDAR survey $(2\times 2)\,\mathrm {m ^ {2}}$ of the surface of the hill above the mine obtained from the Ministry of the Environment and Land and Sea Protection^24^. The simulated transmission $t_ {simu} (\varphi , \theta , \rho )$ can be obtained in the same way as from equation (1) using, in this case, the simulated target and free-sky fluxes:

3 $$\begin{aligned} \Phi _{simu-target}(\varphi ,\theta ,\rho )= & {} \int _{E_{min}(\varphi ,\theta ,\rho )}^{\infty }\phi (\varphi ,\theta ,E) \,dE \end{aligned}$$

4 $$\begin{aligned} \Phi _{simu-free}(\varphi ,\theta )= & {} \int _{E_{0}}^{\infty }\phi (\varphi ,\theta ,E) \,dE \end{aligned}$$

where $E_ {0} \simeq 130\, \textrm{MeV}$ is the minimum muon energy necessary to traverse the detector materials^12^, $E_ {min}$ is the minimum energy for which, in the target measurement, the muon can traverse the target and be detected. This energy value derives from a calculation that takes into account the thickness and density of the materials crossed $l(\varphi , \theta )$. The differential flux model of cosmic muons on the ground $\phi (\varphi ,\theta ,E)$ is obtained from measurements with the ADAMO magnetic spectrometer (INFN Florence)^25,26^. Figure 4d shows the simulated muon transmission obtained assuming a homogeneous density of the target of $2.65 \, \mathrm {g / cm ^ {3}}$ (density of the standard rock^27^). Concerning this last plot, the muons are simulated with a larger acceptance than that of the detector. The absence of events in the NE direction near the boundary (for $\theta \sim 60^{\circ }$-$70^{\circ }$) is due to the significant overburden of the rock in that region due to the presence of the hill. Also, the presence in the centre of the map (in the vertical direction) of a high transmission zone, is due to the fact that the LiDAR data accurately portrays the area of the collapse of the Gran Cava and the main entrance and it is included in the simulated target.

**Figure 5:**
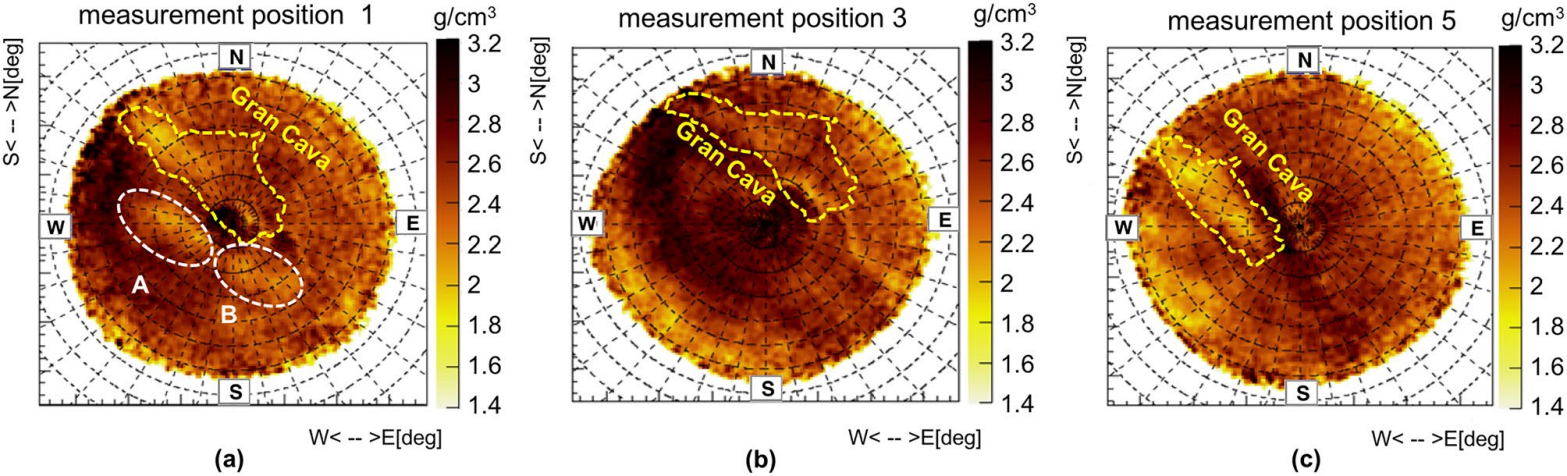
The two-dimensional average density angular distributions obtained from measurement positions 1, 3 and 5. The images are obtained with the ROOT framework.

Simulations are also key to obtain an average density distribution from the transmission angular distribution as described in the introduction. Figure 5a shows the two-dimensional angular distributions of the average density obtained from the three measurement positions (with the Gran Cava and two previously unknown cavities indicated with A and B) in range of zenith angles within about $60^{\circ }$. It is observed that the Gran Cava profile corresponds to zones whose density is less than 2-$2.2\,\mathrm {g/cm^{3}}$. These values are lower than the densities of the typical rocks that can be found in the mine (some of which are shown in Table 1) and therefore can be associated with the presence of a cavity.

**Table 1:** Laboratory measured density values ($\rho _{meas}$) of rock samples taken in the Temperino tourist tunnel.

Rock	${\rho _{meas}}$ ${(g/cm^{3})}$
Acid porphyry	$2.41\pm 0.07$
Mafic porphyry	$2.62\pm 0.08$
Skarn	$3.08\pm 0.07$
Marble	$2.70\pm 0.06$

Other areas with lower average densities appear in the plots: some of them correspond to known cavities and tunnels but others, such as those named A and B in Fig. 5a, correspond to cavities that have never been mapped before. The high density values present in the Gran Cava area (near the two surface accesses), indicate the presence of skarn material below the cavity compatible with the values listed in Table 1. The same procedure was used to obtain the density distributions from measurements 3 and 5, which are shown in Fig. 5b,c. In the next section, the three-dimensional imaging reconstruction of the cavities will be described.

### Three-dimensional reconstruction of cavities

#### Triangulation technique for three-dimensional low density areas localization

**Figure 6:**
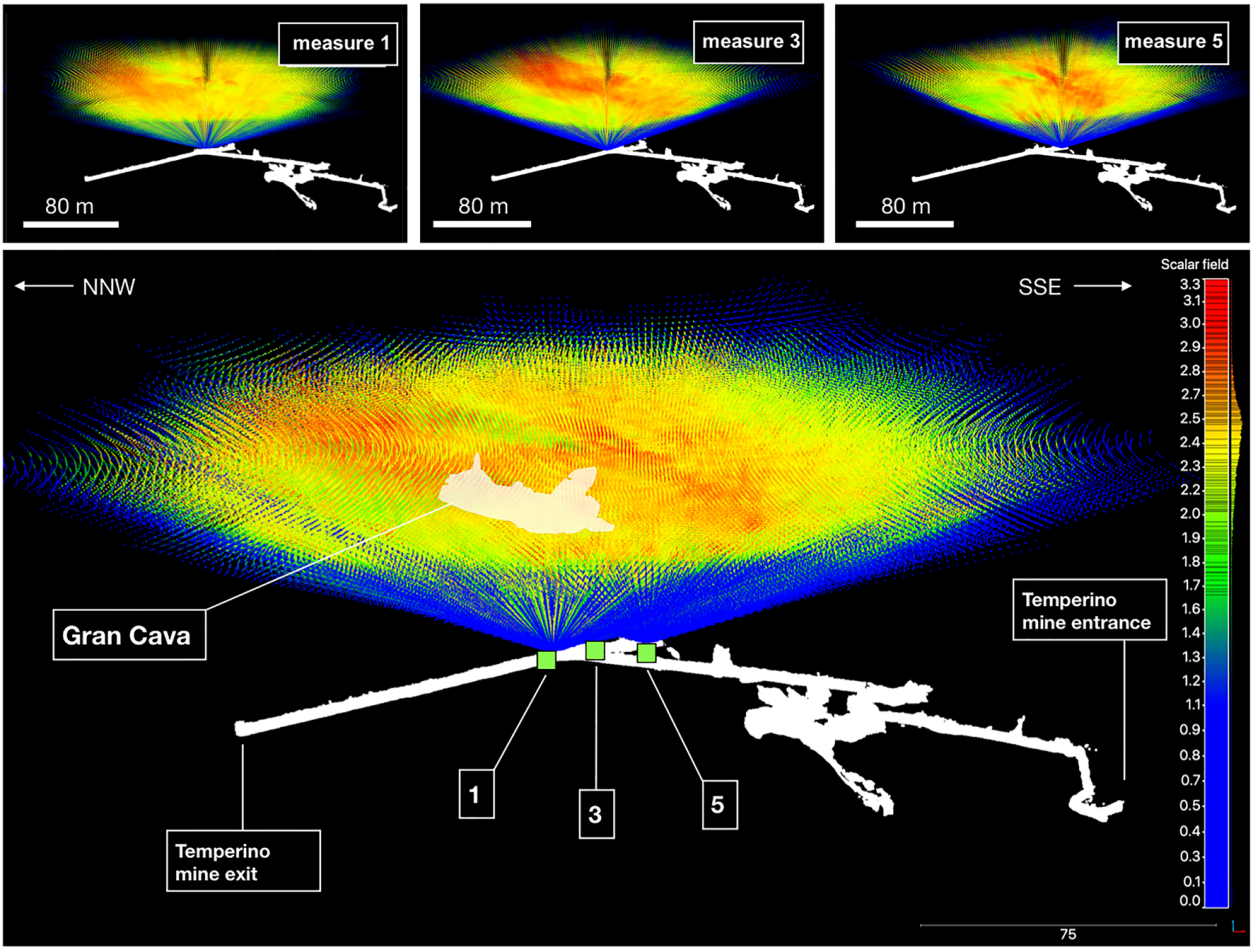
Operating scheme of the triangulation technique applied to the Temperino mine from the three measurement positions (1, 3 and 5) . The images were made with the CloudCompare software. The color scale follows that of the density maps.

Triangulation requires two measurements of the same target with the detector placed in different positions. In this case, the two-dimensional angular density maps obtained from measurements position 1, 3 and 5, shown in Fig. 5, were used. A three-dimensional representation of the principle of operation is shown in Fig. 6. The three cones centred at each measurement point respectively, represent the three angular regions observed by the detector. The rays projecting from the measurement points have a colour scale linked to the average densities observed in that direction. For example, areas in space where low density rays from different measurement positions intersect will correspond to low density objects in space. In the procedure, a three-dimensional world volume was created and divided into cubic voxels with a side of 1 m^23^.

A single voxel’s centre is observed with different polar angles from each of the three measurement positions: $(\varphi _{1},\theta _{1})$, $(\varphi _{3},\theta _{3})$, $(\varphi _{5},\theta _{5})$. If the three sets of coordinates pointing to the voxel correspond to three low density areas in the respective density maps, the voxel is associated with a cavity.

**Figure 7:**
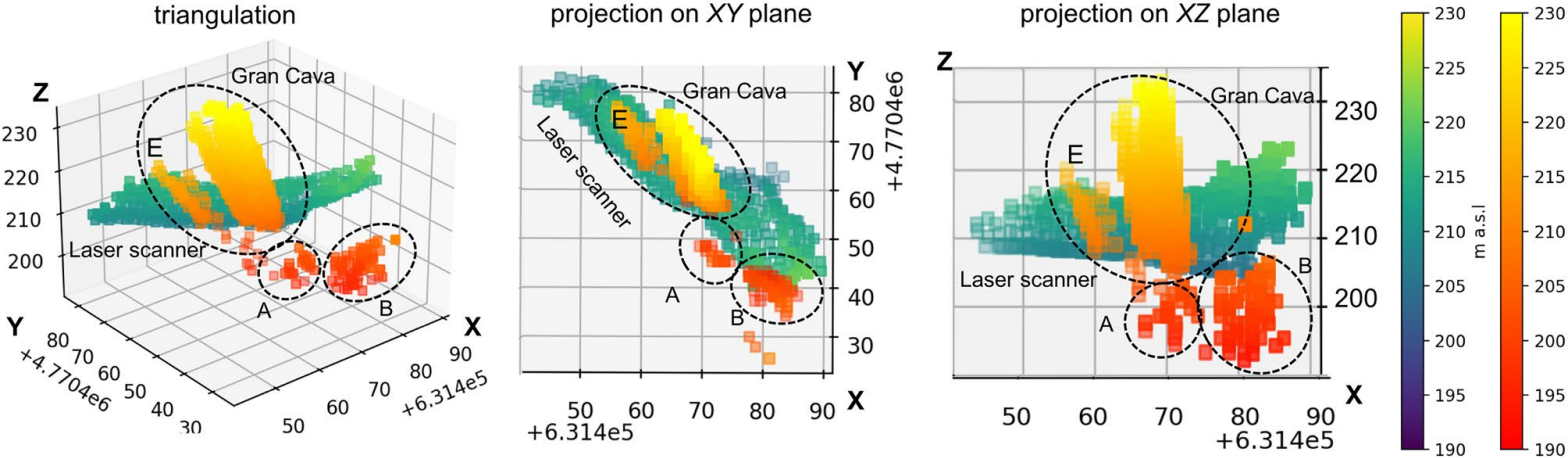
Results of the triangulation technique applied to the three density maps: 3D visualization (left), and the projections on plane *XY* (centre) and *ZX* (right). The colors indicate the altimetry (the orange scale for triangulation results and the blue-green scale for the Gran Cava laser scan) and the *x* and *y* coordinates are reported in the reference system EPSG:32632. The detector is located at about 185 m a.s.l.. The biggest area is identified with the Gran Cava at a relative altitude of about 20 m. The cavities A and B are not present in the cartography’s map of the mine.

The result of the procedure is shown in Fig. 7 in orange scale, where only the voxels which density below a certain threshold are shown. Three distinct clusters emerge: the largest is located at a vertical distance of about 20 m from measurement position 1 (which is located at 185 m above sea level), and is compatible with the Gran Cava, the other two smaller are localized about 15 m (cavity A) and 7 m (cavity B) distance in the vertical direction. Cluster E even though it appears separated from the Gran Cava cluster, is in reality associated to it, the separation beeing an artifact of triangulation method. In fact reconstruction of a cavity based on triangulation of 2D density distributions obtained from different measurement positions requires an intersection of the low density regions independently identified from the different density distributions. This can be significantly affected by the difference in perspectives under which the distribution of the materials present along the line of sight is seen. The same figure shows the point cloud of the Gran Cava obtained with the laser scanner in order to have a visual comparison with its real development. The elongated shape obtained for the Gran Cava is not an accurate depiction of its real spatial development. In fact, this is a limitation of the triangulation technique that is evident whenever the measurement positions are too close to each other, resulting in a loss of vertical resolution and vertical scale dilation.

Clusters A and B, that are identified as cavities, are not found in cartography of the Temperino mine, so we can’t compare the result with their real spatial development. These results anyway triggered first explorations performed by a team of speleologists, that seem to confirm their presence. Unfortunatlelly the entrances are obstructed by cave-ins and mud. The back-projection algorithm can address some of the issues and give a more truthful rendering of the cavity volumes.

#### Back-projection technique, an innovative three-dimensional cavity reconstruction algorithm

**Figure 8:**
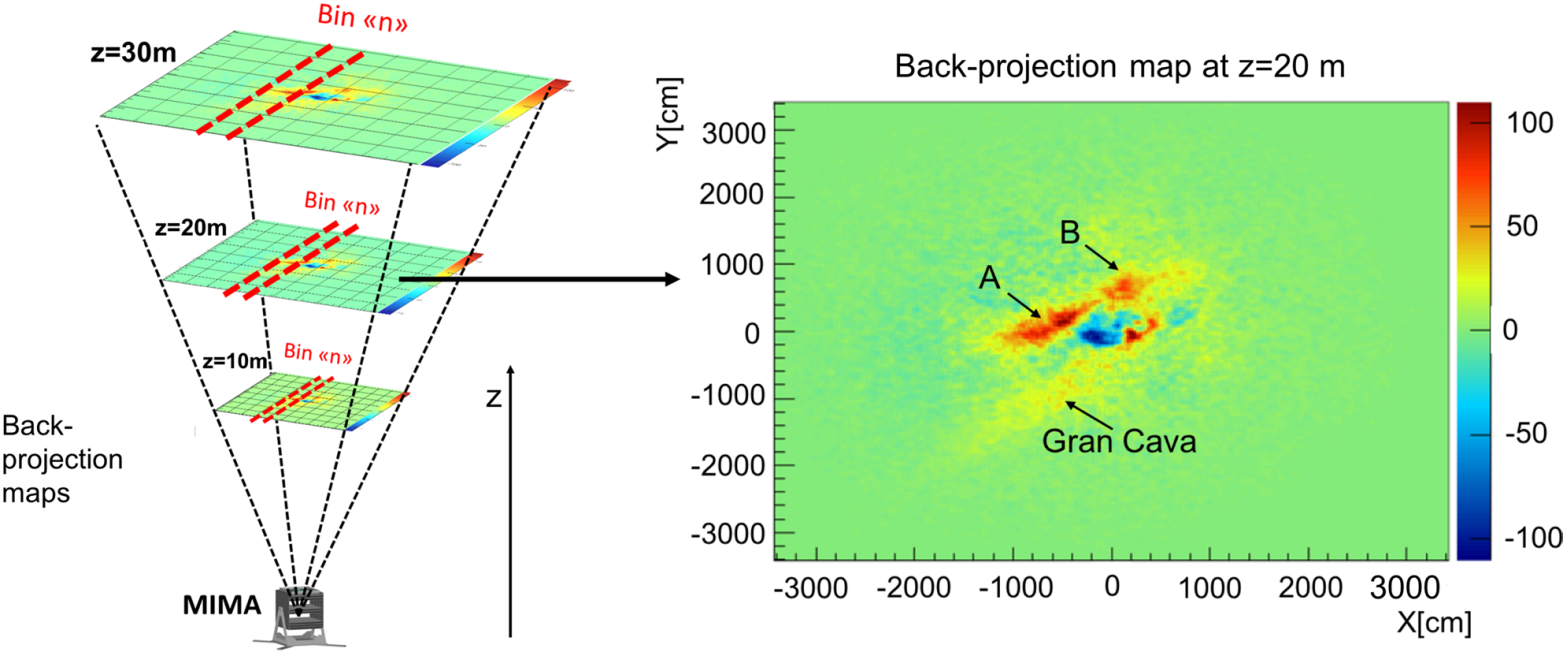
On the left a schematization of the principle used to realized the back-projection maps for different *z*. On the right an example of back-projection map at $z=$20 m (the meaning of the color scale is explained in the text). Three signals are visible: Gran Cava, signal A and B, already observed in the density map. The map is obtained with the ROOT framework.

The back-projection technique was applied at the Temperino mine to estimate the distance from the detector to the cavities and their profiles as seen by the detector^23^. Only the data acquired from the measurement position 1 were used. An example of a back-projection map created at a relative altitude of $z=20$ m with respect to the detector, is shown in Fig. 8 on the right. The Cartesian axes represent the linear size of the back-projection plane at the *z* level. Every back-projection map is a two-dimensional histogram created at different values of the *z* coordinate in space and is centered on the vertical direction of the detector. A schematic representation is shown in Fig.8 on the left. Each bin contains the difference between the number of back-projected tracks in the target and in the free-sky configuration, normalized using the simulated transmission. Three compact zones with a high number of tracks corresponding to the three cavities are shown. Compared to density maps, back-projection maps have an inverted *Y* axis because they are displayed in the detector reference system.

**Figure 9:**
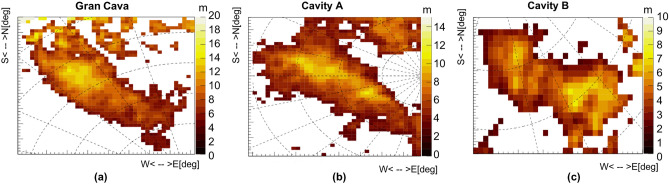
Two-dimensional cavity air thickness maps for the Gran Cava, and A and B signals. The images are obtained with the ROOT framework.

The back-projection technique was applied to each single cavity signal, obtaining a three-dimensional bottom profile of the cavities as seen from the detector’s point of view. In particular, to estimate the profiles of the observed cavities, the technique was applied to slices of the signal (as shown schematically by the red lines in Fig. 8) with a width of 30 cm at a distance of 20 m. This choice defines the accuracy of the reconstruction on the horizontal direction and takes into account the angular resolution of the detector, the presence of multiple scattering and muon statistics in a single slice. To create a three-dimensional rendering of the Gran Cava and the other two cavities, point clouds of the bottom profiles were created and were interpolated approximating the measured profiles. For the three identified low density areas, that we assumed corresponding to cavities, we estimated the air thickness in the different directions based on the average density values along line of sight that characterize the cavities and the surrounding rocks. Figure 9 shows the air thickness angular distributions for the three cavities. With this method a Gran Cava thickness results of the order of 12-14 m was obtained, comparable to the laser scan survey values. For cavities A and B the thickness of the order of 8 m (for A) and 6 m (for B). Starting with the bottom profile obtained with the back-projection technique, adding the estimated air thicknesses, a three-dimensional reconstruction of the entire low density volumes was obtained.

**Figure 10:**
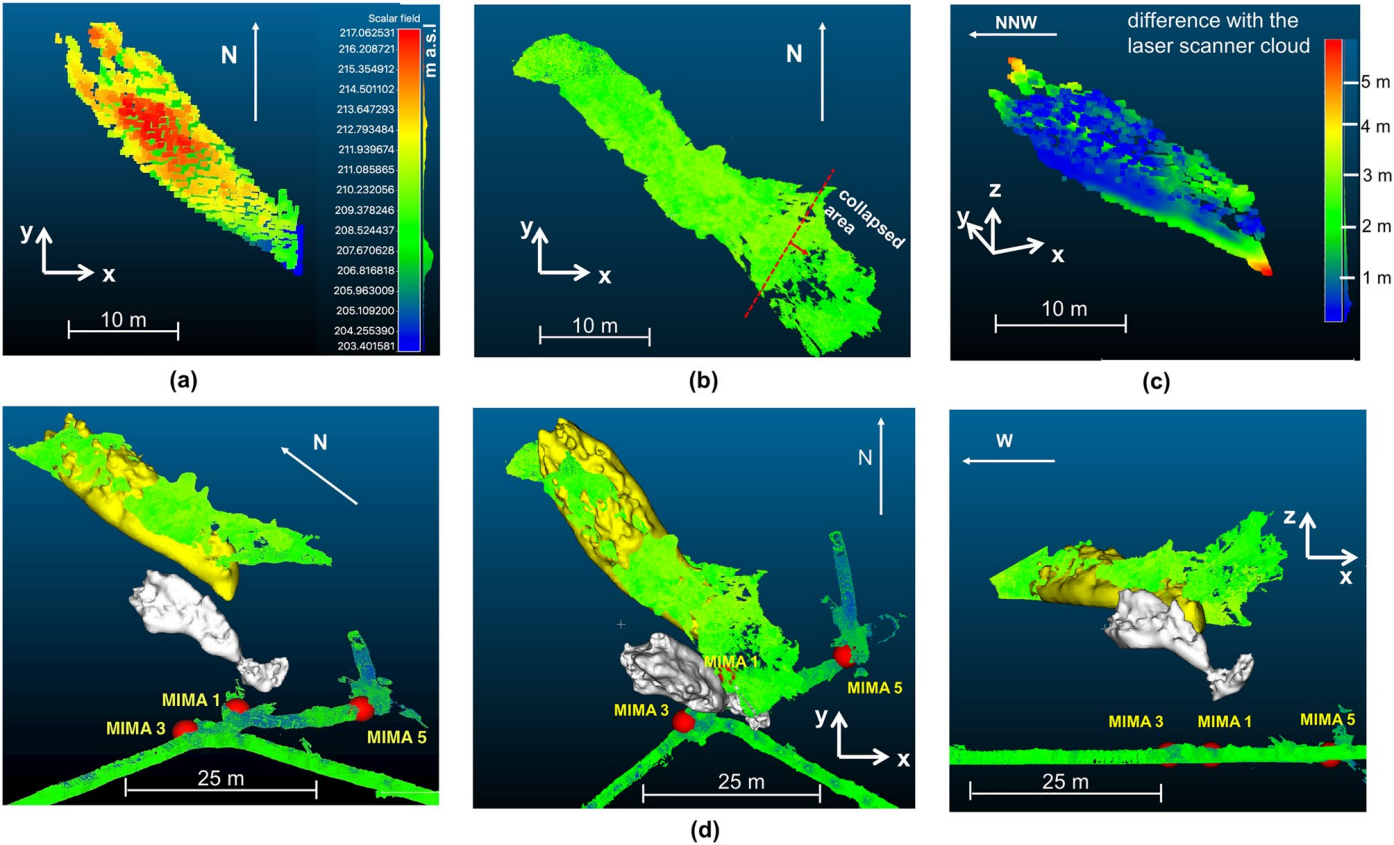
Three-dimensional representation of reconstructed cavities using the back-projection technique. In (**a**) the point clouds relating to the Gran Cava (on the left) reconstructed exclusively with the muographic measurements (in altitude colour’s scale) is shown. In (**b**) the point cloud of the Gran Cava acquired with the laser scanner technique is shown. In (**c**) there’s the difference between the Gran Cava point cloud obtained with the muon radiography technique and that obtained with the laser scanner. The difference was evaluated with the CloudCompare software. In (**d**) the three cavities observed, the laser scanner point cloud of the tourist tunnel, and the three points of MIMA measurement positions (drawn as spheres) are shown with mixed, top and lateral orientations.

Figure 10a shows the reconstructed air volume of the Gran Cava using the procedure described above, while Fig. 10b the laser scanner point cloud. The laser scan also contains a region extending in the SE direction that corresponds to a region of the Gran Cava where the vault has collapse and is visible from the exterior. The images were visualized using the CloudCompare software and show a substantial overlap. To have a quantitative estimate of the difference between the two point clouds, the method “Cloud-to-cloud distance” implemented in the CloudCompare software was used. The method calculates the Euclidean distance between each point of the laser scan point cloud (point cloud of Fig. 10b) respect to the closest point of the muographic point cloud (point cloud of Fig. 10a). The distance thus calculated is displayed as a color scale on the muographic point cloud. The result is shown in Fig. 10c. The average difference measured between the two point clouds is nearly everywhere less than one metre. There are some differences in the vault collapsed area and in the volume corresponding to the entrance. The slope in the collapse area seen with muon radiography is steeper than that seen with the laser scanner, probabily due to the presence of many small boulders on the floor that can hide the bottom floor from the laser scanner itself. In the North direction, near the Gran Cava entrance, there is an area that has not been mapped with the laser scanner and which could introduce some uncertainties in the simulated terrain surface profiles. Other small cavities or tunnels partly buried in that region can entangle their signals with the one linked to the Gran Cava. The three-dimensional reconstruction has been repeated for cavities A and B. Figure 10d shows all three cavities three-dimensional meshes as reconstructed from muographic data.

**Figure 11:**
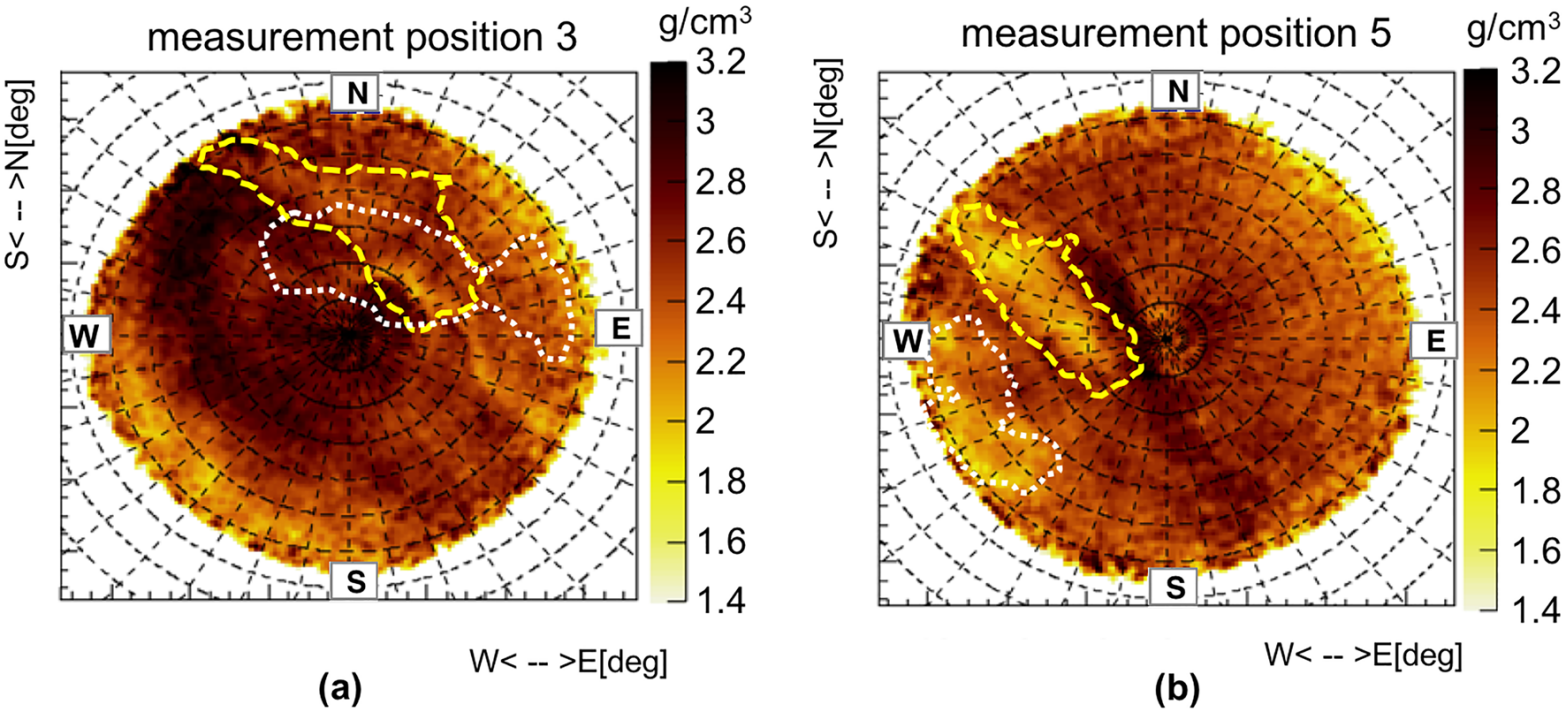
The mesh profile of unknown cavities reconstructed from measurement 1 superimposed on the density maps of the other measurements. In (**a**) and (**b**) the density angular distribution relating to the measurement positions 3 and 5 on which the profile of the reconstructed mesh is superimposed: the Gran Cava (dashed line), A and B (dotted line). The images are obtained with the ROOT framework.

Finally, a consistency test was performed: the mesh profiles of the unknown cavities reconstructed from measurement position 1 were overlapped on the density maps obtained from the other measurement positions (Fig 5b,c). If the shape, size and height of reconstructed meshes from measurement 1 alone are realistic, their profiles should overlap with low density zones in these maps. The results are shown in Fig. 11 in which an overlap of these profiles with areas of lower density is visible. While for measurement position 5 the correspondence with areas at low density areas is evident, for measurement position 3 it is less clear. This is probably due to high density material which is partly shadowing the signal of the Gran Cava quarry along the line of sight from this measurement position.

## Conclusion

Muographic measurements were carried out at the Temperino mine, in Tuscany (Italy), to verify the technique’s potential for the discovery and mapping of cavities and ore bodies. The results shown in this paper focus on the localization and three-dimensional reconstruction of empty cavities. The identification of existing tunnels, passages or caves is of considerable importance both for safety^28^ reasons and for the development of ancient abandoned mines as tourist attraction. In our case, given the history of the Temperino complex, there is also a considerable interest in the field of archaeology.

Three-dimensional reconstruction algorithms were applied to the low density anomalies observed in the density maps obtained from muographic measurements in a specific area of the Temperino mine. Through triangulation, with data obtained from three different measurement positions, a first estimate of the localization in space of the low density regions was obtained. The results are compatible with the position of a know quarry (i.e. Gran Cava) and suggest the existence of two previously unknown cavities not present in the cartographic maps of the mine. Comparing the three-dimensional development of the known cavity with its real development obtained with the laser scanner, some limits of the triangulation technique were observed, in particular the elongated vertical shape of the reconstructed volumes when the measuring positions are too close. Finally, an innovative algorithm that uses only the data obtained from a single muographic measurement, and that is based on the back-projection technique, was developed. Using this new tool, the volumes of the Gran Cava and the hitherto unknown cavities were reconstructed with a resolution of less than one metre. A comparison with the laser scan profile of the Gran Cava was made, indicating a substantial agreement with the reconstructed volume. A further consistency check was made using the data from the other two measurement positions, where it was found that the 3D-mesh profiles obtained with the back-projection algorithm, correspond to low-density zones in these maps. These results not only confirm the applicability of muography as a geological prospecting tool but also its reliability, opening the way to other possible applications in the archaeological and civil engineering fields.

In the future, more muography measurements are planned at the Temperino mine and at the Collins mine (LI, Tuscany, Italy) in order to evaluate the health and safety issues (e.g. radon infiltration through old and partially filled excavation pits) of some of the areas that will be made available for tourist visits.

## Data Availability

The datasets analyzed during the current study are available from the corresponding author on reasonable request.
